# Supplementary material for: Human cytomegalovirus evades antibody-mediated immunity through endoplasmic reticulum-associated degradation of the FcRn receptor
Source: Nat Commun. 2019 Jul 9;10:3020. doi: 10.1038/s41467-019-10865-y (PMC6617459; doi:10.1038/s41467-019-10865-y)
Supplement: Supplementary file 1 — Supplementary Information [file 41467_2019_10865_MOESM1_ESM.pdf]

Human cytomegalovirus evades antibody-mediated immunity through endoplasmic reticulum-associated degradation of the FcRn receptor

Liu et al.

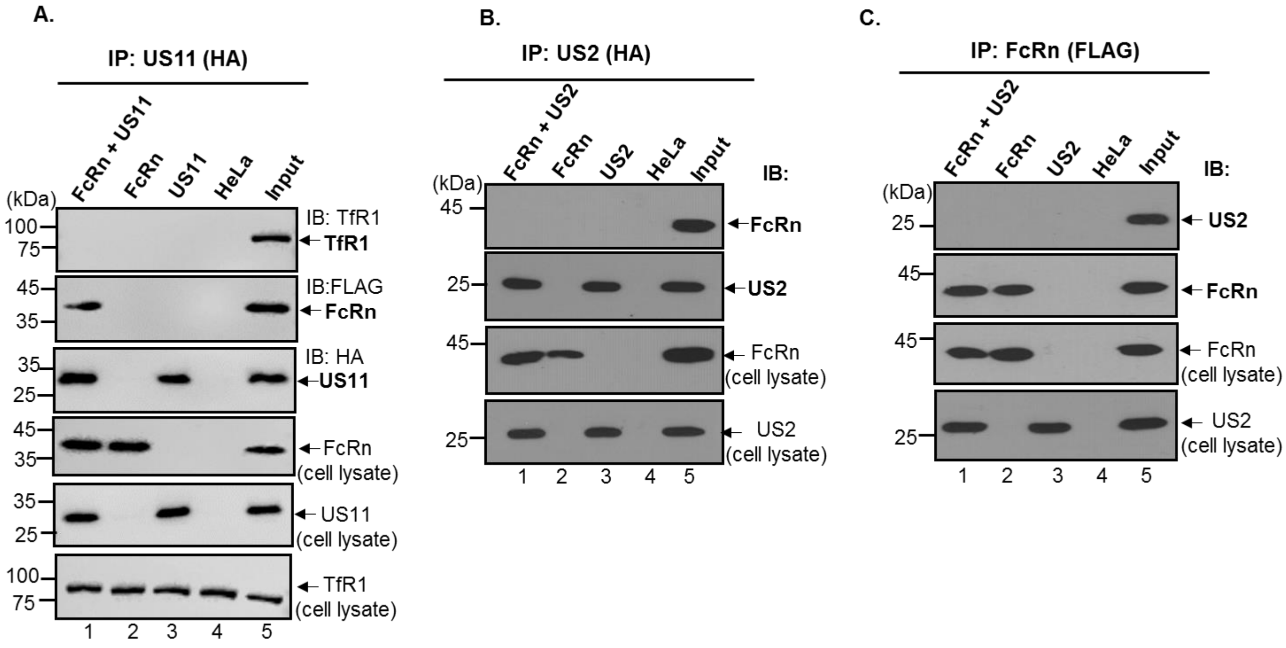

Supplementary Figure 1. US11 does not interact with endogenous transferrin receptor 1 (TfR1) and FcRn does not interact with the HCMV US2. ***A.*** The cell lysates from $HeLa^{FcRn+US11}$ (lane 1), $HeLa^{FcRn}$ (lane 2), $HeLa^{US11}$ (lane 3), and HeLa control (lane 4) were immunoprecipitated by mAb anti-HA for US11. ***B-C.*** The cell lysates from $HeLa^{FcRn+US2}$ (lane 1), $HeLa^{FcRn}$ (lane 2), $HeLa^{US2}$ (lane 3), and HeLa control (lane 4) were immunoprecipitated by mAb anti-HA for US2 or anti-FLAG for FcRn. The immunoprecipitates were subjected to 12% SDS-PAGE electrophoresis under reducing conditions, then transferred to a nitrocellulose membrane for Western blotting with anti-TfR1, anti-FLAG (FcRn), or HA (US11) mAb as indicated. The 50 mg cell lysates (input) were blotted with the indicated Abs. The location of the TfR1, FcRn HC or US2 is indicated by an arrow. Source data are provided as a Source Data file.

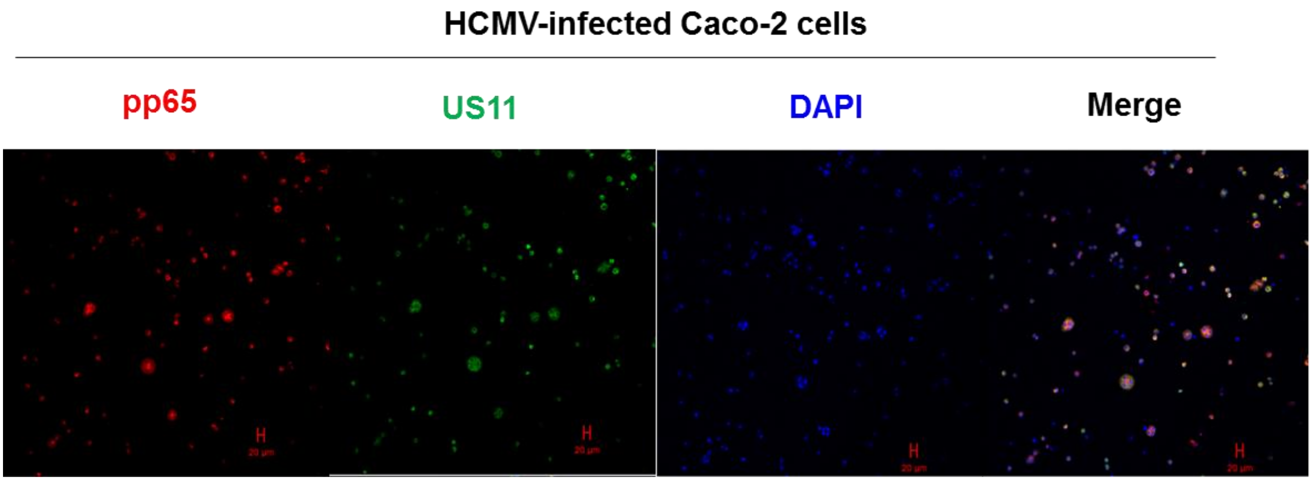

Supplementary Figure 2. HCMV-infected Caco-2 cells. Caco-2 cells were grown on glass coverslips and infected with HCMV at an MOI of 5. At day 2 p.i., monolayers were fixed with 4% paraformaldehyde and permeabilized in 0.2% Triton X-100. Subsequently, the cells were incubated with affinity-purified anti-US11 (green) or anti-pp65 (red) specific Ab, followed by Alexa Fluro 488- or 555-conjugated IgG. Staining that appears yellow in the merged images indicates colocalization of US11 with pp65. The nuclei were stained with DAPI (blue). Scale bar: 20μm.

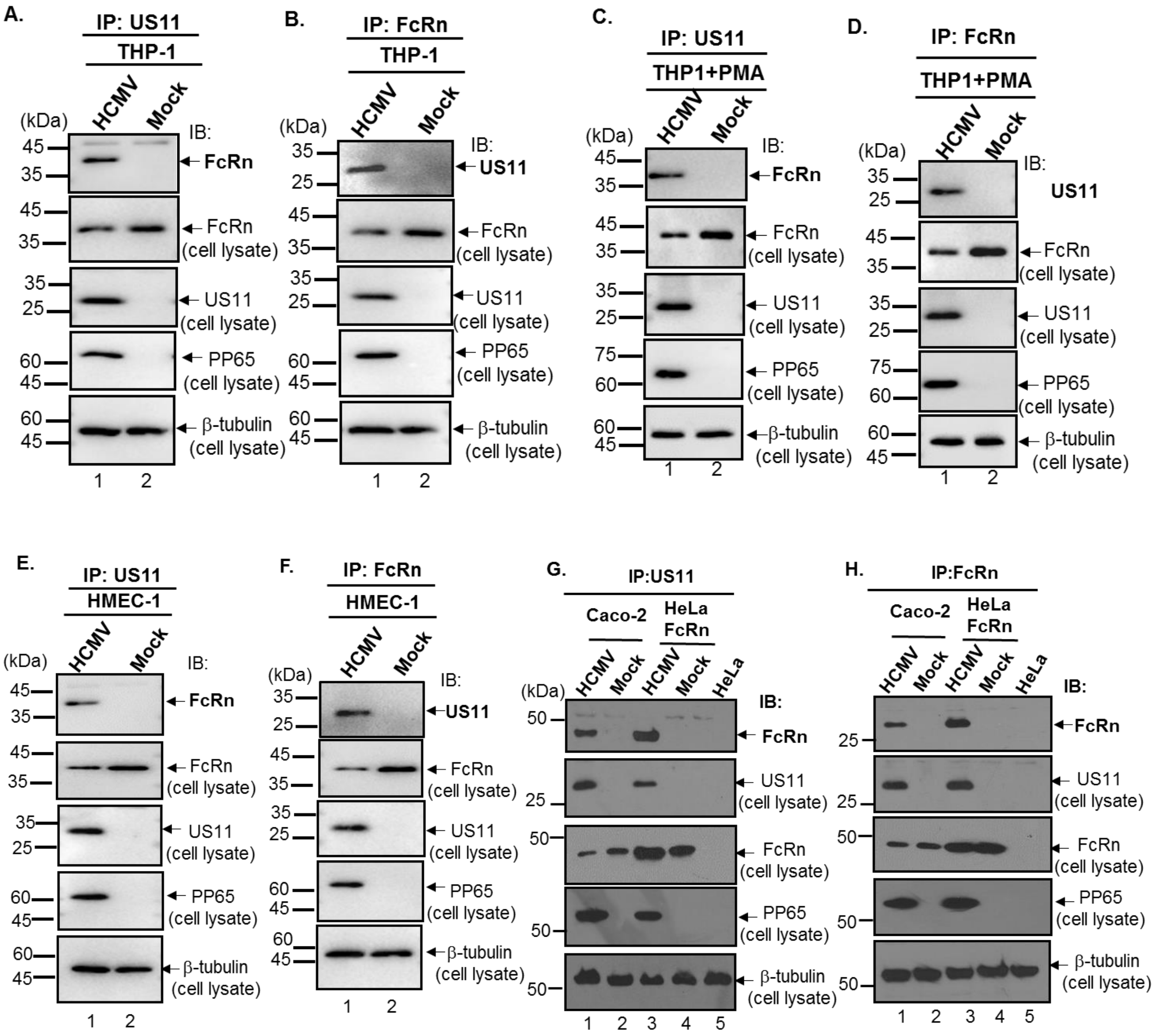

Supplementary Figure 3. FcRn interacts with US11 in HCMV-infected THP-1, HMEC-1, and Caco-2 cells. THP-1 cells were treated with 50 ng/ml PMA or left untreated for 48 hrs. THP-1 cells (***A,D***), HMEC-1 cells (***E,F***), and Caco-2 cells (***G,H***) were mock-infected or infected for 24 hrs with clinical strain HCMV at an MOI of 5. $HeLa^{FcRn}$ and HeLa cells were used as controls (***G,H***). The cell lysates were immunoprecipitated by US11 or FcRn specific Abs. The immunoprecipitates were subjected to 12% SDS-PAGE electrophoresis under reducing conditions, then transferred to a nitrocellulose membrane for Western blotting with anti-US11 or FcRn as indicated. Immunoblots (IB) were developed with ECL. The 20 μg cell lysates (input) were blotted with the indicated Abs. pp65, an HCMV major tegument protein, is used for monitoring viral infection. The location of the proteins is indicated by an arrow. Source data are provided as a Source Data file.

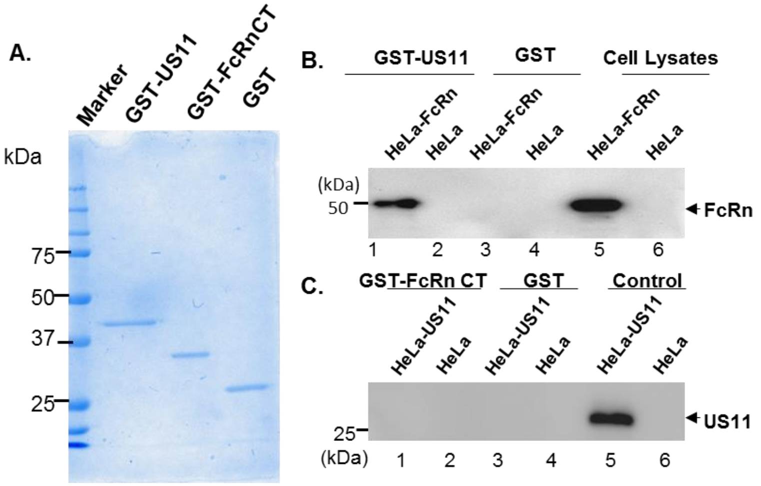

Supplementary Figure 4. US11 interacts with FcRn through its ER-luminal domain. The cDNA fragment encoding extracellular domain of US11 or cytoplasmic tail of FcRn was fused to the GST and expressed as a GST fusion protein. Productions of GST fusion proteins are described in *Materials and Methods*. ***A.*** GST, GST-US11, and GST-FcRn CT fusion proteins were stained with Commassie blue and used for in vitro pull-down assays. ***B.*** GST-US11 proteins were incubated with the cell lysates from HeLa^{FcRn} (lane 1) or FcRn-negative HeLa (lane 2) cells. GST proteins are shown as negative controls in lanes 3, 4, respectively. Cell lysates are used as loading control (lanes 5, 6). ***C.*** FcRn cytoplasmic tail (CT) expressed as a GST fusion protein were incubated with HeLa^{US11} (lane 1), HeLa (lane 2). GST protein is a control in lanes 3 and 4. HeLa^{US11} or HeLa cell lysates are used as loading control (lanes 5, 6). Beads were completely washed with buffers. In each experiment, GST-fusion protein binding was assessed by immunoblot as indicated. We found that GST-US11, captured human FcRn (***B***) from HeLa^{FcRn} cells, while a GST fusion protein containing only the cytoplasmic tail of FcRn failed to pull down US11 from HeLa^{US11} cells (***C***). The results support our hypothesis that the main site of contact between US11 and FcRn is between their extracellular domains. Source data are provided as a Source Data file.

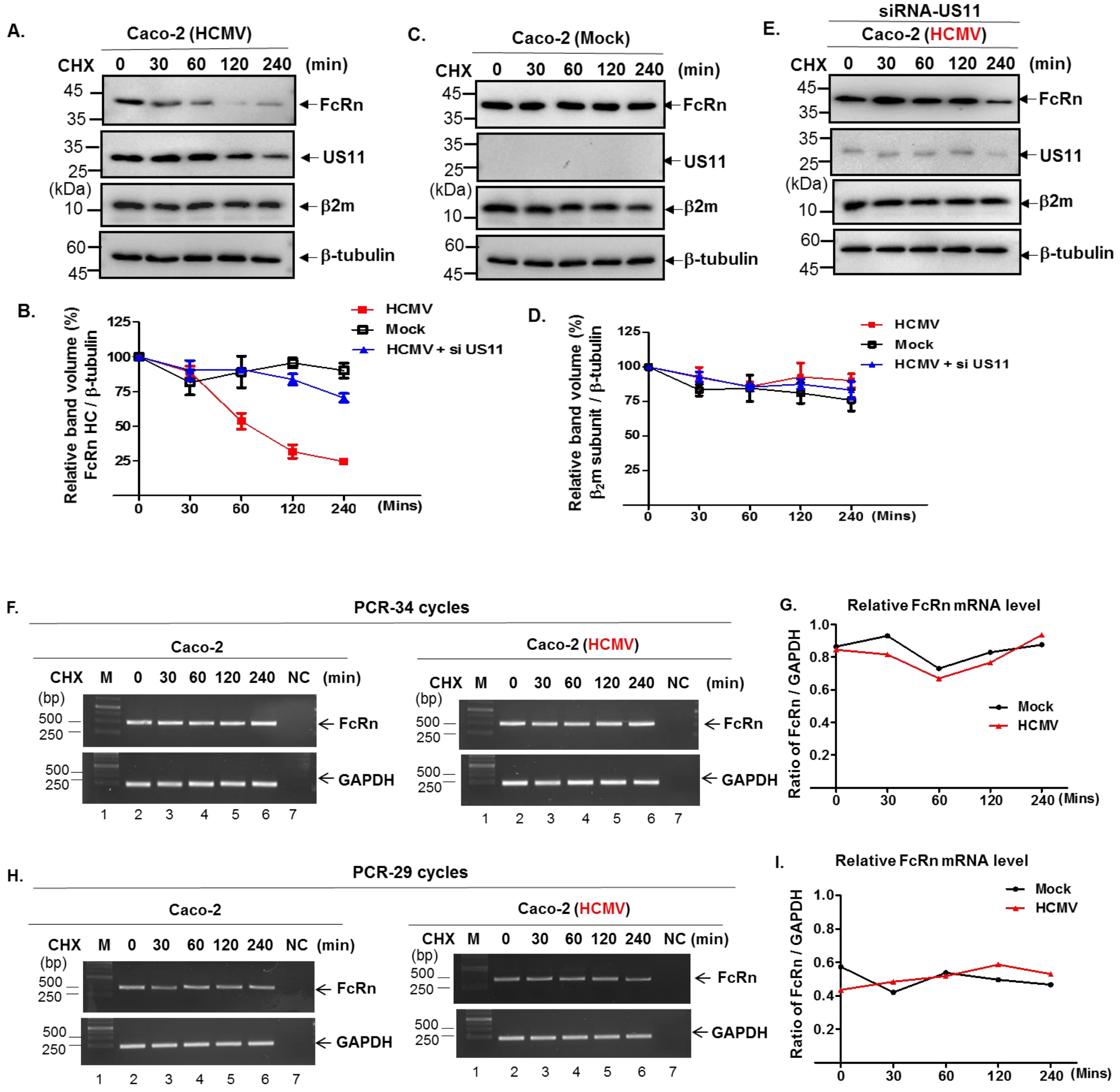

Supplementary Figure 5. Time course effects of HCMV infection on FcRn protein and mRNA expression. ***A-E.*** Caco-2 cells were infected with clinical strain HCMV (MOI 5) (***A***) or mock-infected (***C***) for 48 hr. The infected cells were also transfected with 20 nM US11 siRNA oligomers (***E***). 48 hr later, cells were then treated with CHX (100 µg/ml) for the indicated time. The cells were lysed after CHX treatment and cell lysates (20 µg) were subject to Western blotting with corresponding Abs as indicated. The level of remaining endogenous FcRn (***B***) and β_2m (***D***) at different time points was quantified as the percentage of the β-tubulin level. The percentage of time point 0 (min) is assigned a value of 100% and the values from other time points are normalized to this value. Error bars represented mean ± SEM of three independent repeats. ***F-I.*** Human intestinal cell line Caco-2 was mock infected (*left*) or infected with HCMV (MOI 5, *right*). Total RNA was isolated at the indicated time by TRIzol reagent and analyzed by semiquantitative RT-PCR for FcRn mRNA. GAPDH amplification was used as an internal control. PCR amplifications were run at 34 (**F**, *top*) or 29 (**H**, *bottom*) cycles to exclude the potential saturation of PCR amplification. The relative FcRn mRNA levels (***G*** or ***I***) were calculated by the ratio of FcRn mRNA levels to GAPDH mRNA levels. The mRNA levels were quantified by the DNA band density (relative band volume) as measured by Image Lab 5.2. The percentage of time point 0 (min) is assigned a value of 100% and the values from other time points are normalized to this value. Source data are provided as a Source Data file.

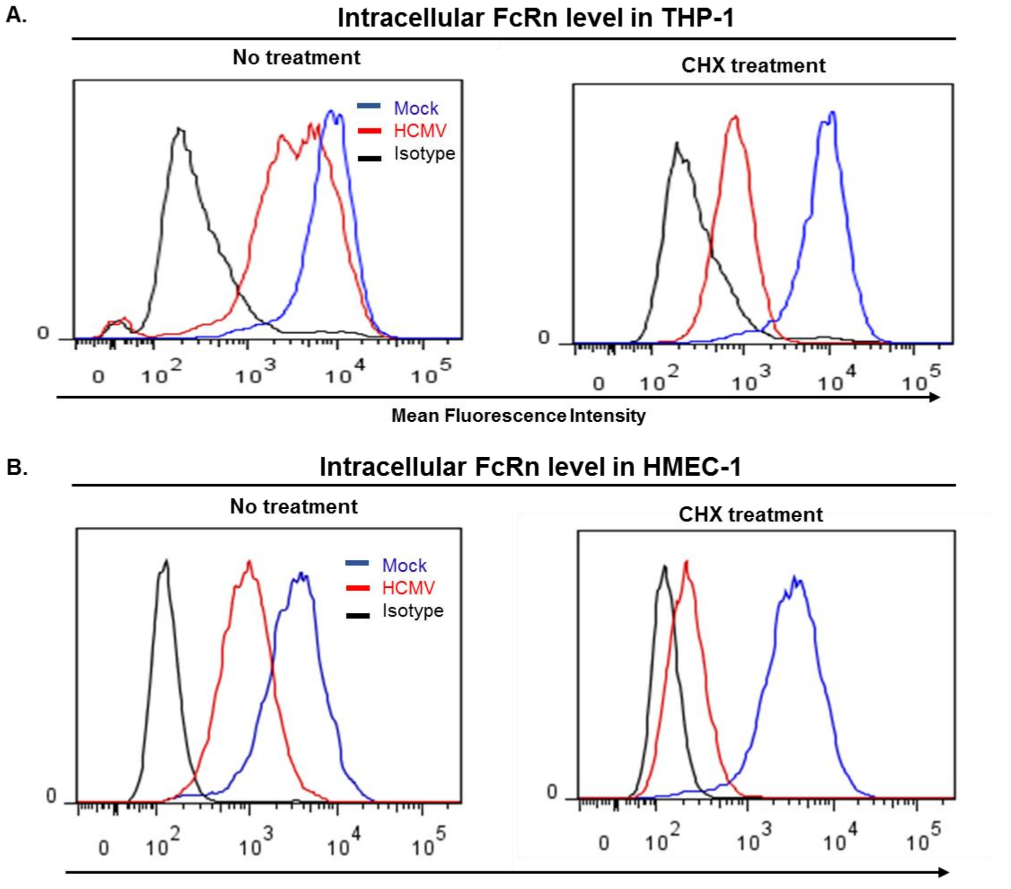

Supplementary Figure 6. Intracellular Expression of FcRn in HCMV-infected THP-1 and HMEC-1 cells. Intracellular expression of FcRn in mock- or HCMV-infected THP-1 (***A***) and HMEC-1 (***B***) cells (10^6) at an MOI of 5 were measured by flow cytometry. 48 hr post infection, the equal number of cells were treated with Cycloheximide (100 μg/ml) or left untreated for 4 hr. Cells were then blocked with 2% FBS supplemented with 30 μg/ml human Fc block and subsequently stained as described in *Materials and Methods*. Results are expressed as histograms of fluorescence intensity (log scale). The red or blue histograms represent staining of cells with anti-FcRn-specific Ab in the presence or absence of HCMV infection, and the black histograms represent cells stained with irrelevant IgG. The staining was conducted three times with similar results. The mean fluorescence intensity (MFI) is shown on the x-axis, and the relative cell number on the y-axis.

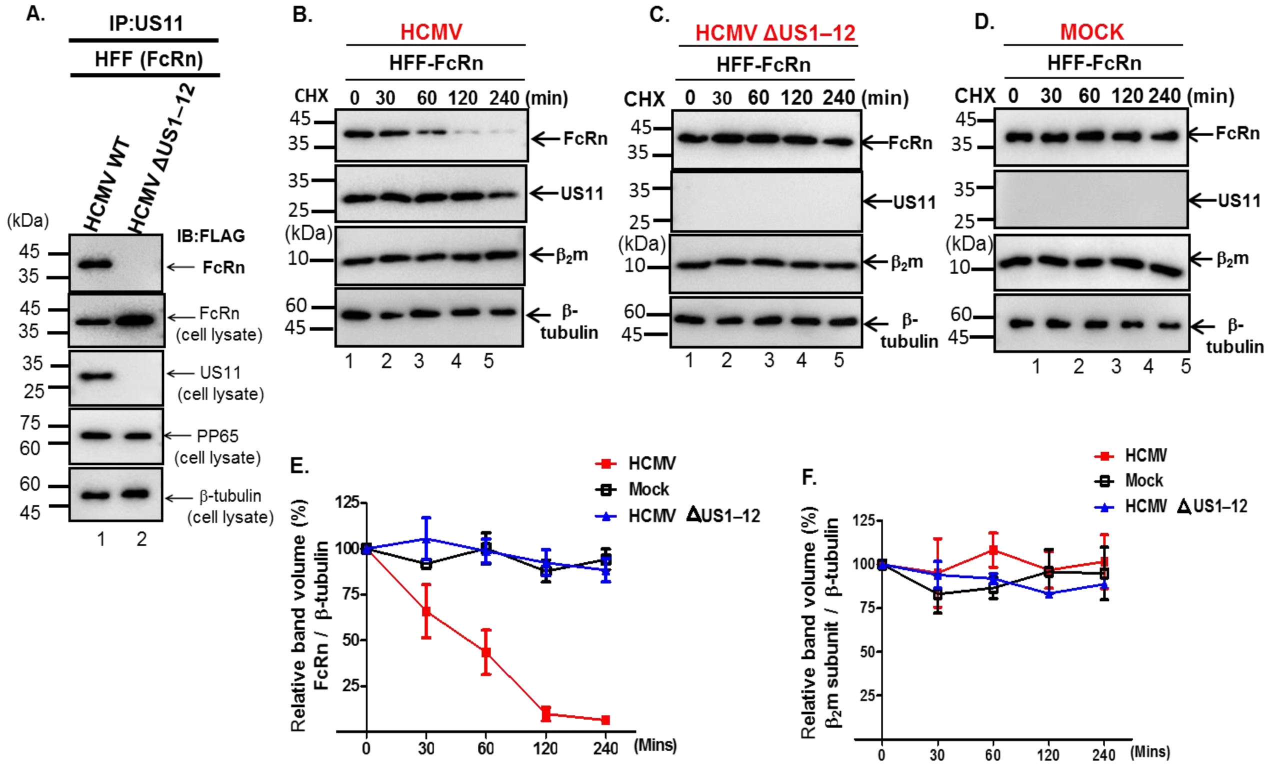

Supplementary Figure 7. HCMV ΔUS1-12 mutant virus fails to cause US11 degradation. ***A.*** Verification of US11 expression in HCMV ΔUS1-12 mutant virus. HFF^{FcRn} cells were infected with HCMV or HCMV ΔUS1-12 virus at an MOI of 5. The infection was verified by detecting HCMV phosphoprotein 65 (PP65). At day 2 p.i., the cell lysates from infected cells were immunoprecipitated with anti-US11 Ab. All immunoprecipitates or cell lysates (20 μg) as internal controls were subject to Western blotting with corresponding Abs as indicated. ***B-E.*** HFF^{FcRn} cells were infected with clinic strain HCMV (MOI 5) (***B***), HCMV ΔUS1-12 (***C***), or mock-infected (***D***) for 48 hr. 48 hr later, cells were then treated with CHX (100 μg/ml) for the indicated time. The cells were lysed after CHX treatment and cell lysates (20 μg) were subject to Western blotting with corresponding Abs as indicated. The level of remaining endogenous FcRn (***E***) and β_2m (***F)*** at different time points was quantified as the percentage of the β-tubulin level. The percentage of time point 0 (min) is assigned a value of 100% and the values from other time points are normalized to this value. Error bars represented mean ± SEM of three independent repeats. Source data are provided as a Source Data file.

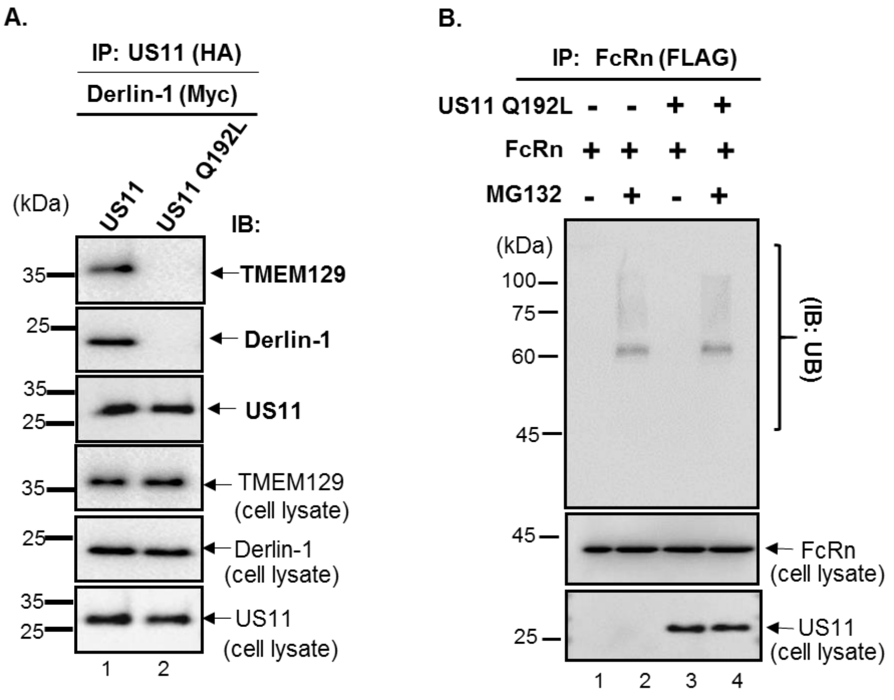

Supplementary Figure 8. The US11 transmembrane domain is required for Derlin-1 interaction and FcRn ubiquitination. ***A.*** The interaction between US11 and Derlin-1 is dependent on a polar glutamine residue in the US11 transmembrane domain. HeLa ^{US11} or HeLa ^{US11Q192L} stable cells were lysed and US11 was immunoprecipitated and eluted in SDS sample buffer. Immune precipitates (top) and total lysates (bottom) were analyzed by SDS/PAGE and probed for TMEM129, Derlin-1, and US11. Derlin-1 and TMEM129 associates with wild-type US11 but association with the mutant US11-Q192L is dramatically reduced. ***B***. US11-Q192L fails to induce FcRn ubiquitination. HeLa^{FcRn} cells were transfected with PEF6 plasmid or pEF6-HA-US11Q192L for 24 hr. Cells were subsequently treated with 50 μM MG132 for 4 hr and then lysed in PBS with 0.5% CHAPS and protease inhibitor cocktail. After immunoprecipitation of FcRn with rabbit anti-FLAG, immunoprecipitated complexes were subjected to SDS-PAGE and analyzed by Western blot with mouse anti-ubiquitin Ab. The cell lysates (20 μg) from each sample were blotted for monitoring the levels of FcRn or US11Q192L expression. Source data are provided as a Source Data file.

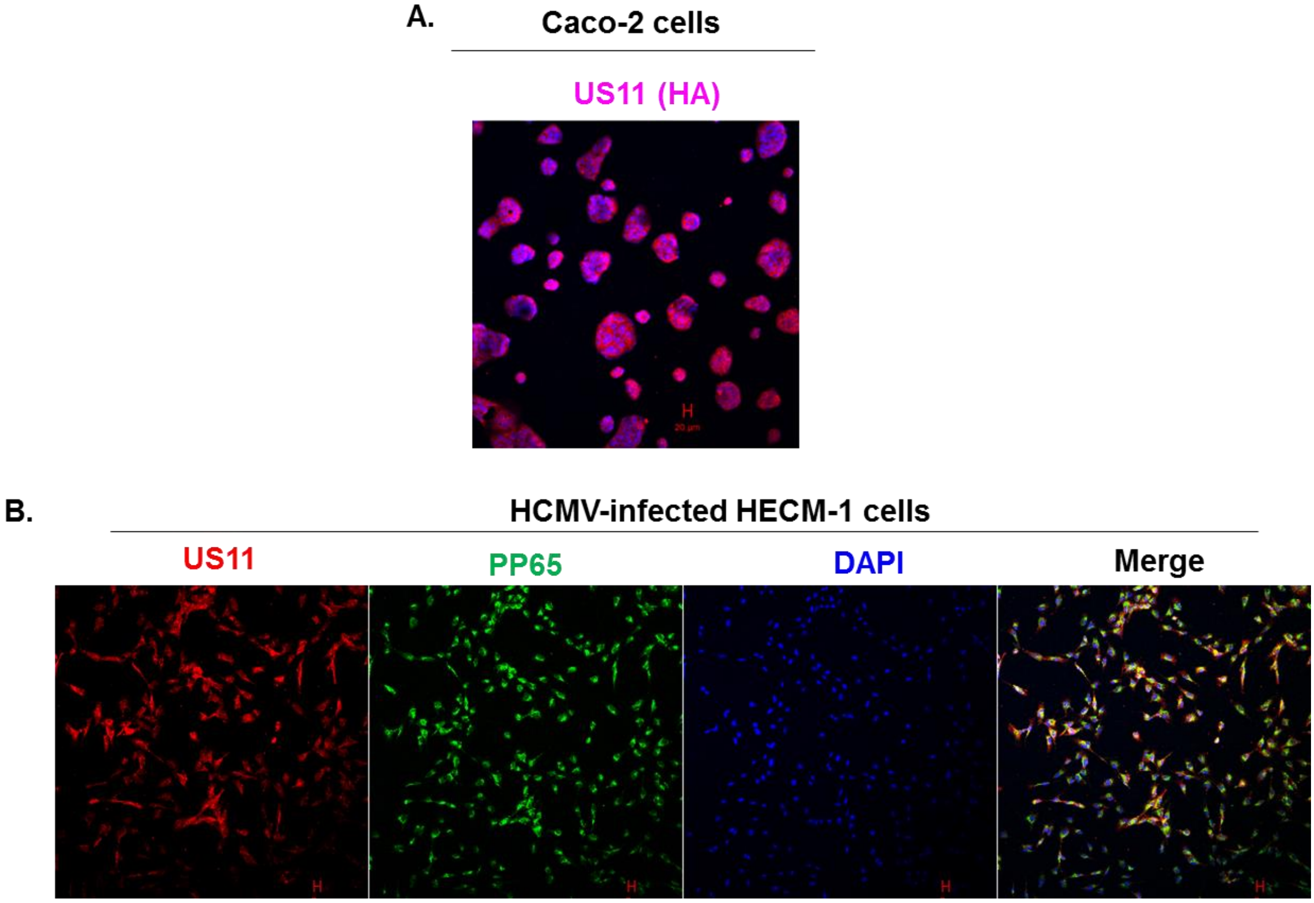

Supplementary Figure 9. Expression of US11 in Caco-2 cells and infection of HECM-1 cells by HCMV virus. ***A.*** Caco-2 cells transfected with a plasmid encoding US11. Caco-2 cells were transfected with US11 plasmid and grown on glass coverslips. 48 hr later, monolayers were fixed with 4% paraformaldehyde and permeabilized in 0.2% Triton X-100. Subsequently, the cells were incubated with HA specific Ab for US11, followed by Alexa Fluro 555-conjugated IgG. The nuclei were stained with DAPI (blue). Scale bar: 20 μm. ***B.*** HCMV-infected HECM-1. HECM-1 cells were grown on glass coverslips and infected with HCMV at an MOI of 5. At day 2 p.i., monolayers were fixed with 4% paraformaldehyde and permeabilized in 0.2% Triton X-100. Subsequently, the cells were incubated with affinity-purified anti-US11 (green) or anti-pp65 (red) specific Ab, followed by Alexa Fluro 488- or 555-conjugated IgG. Staining that appears yellow in the merged images indicates colocalization of US11 with pp65. The nuclei were stained with DAPI (blue). Scale bar: 20 μm.

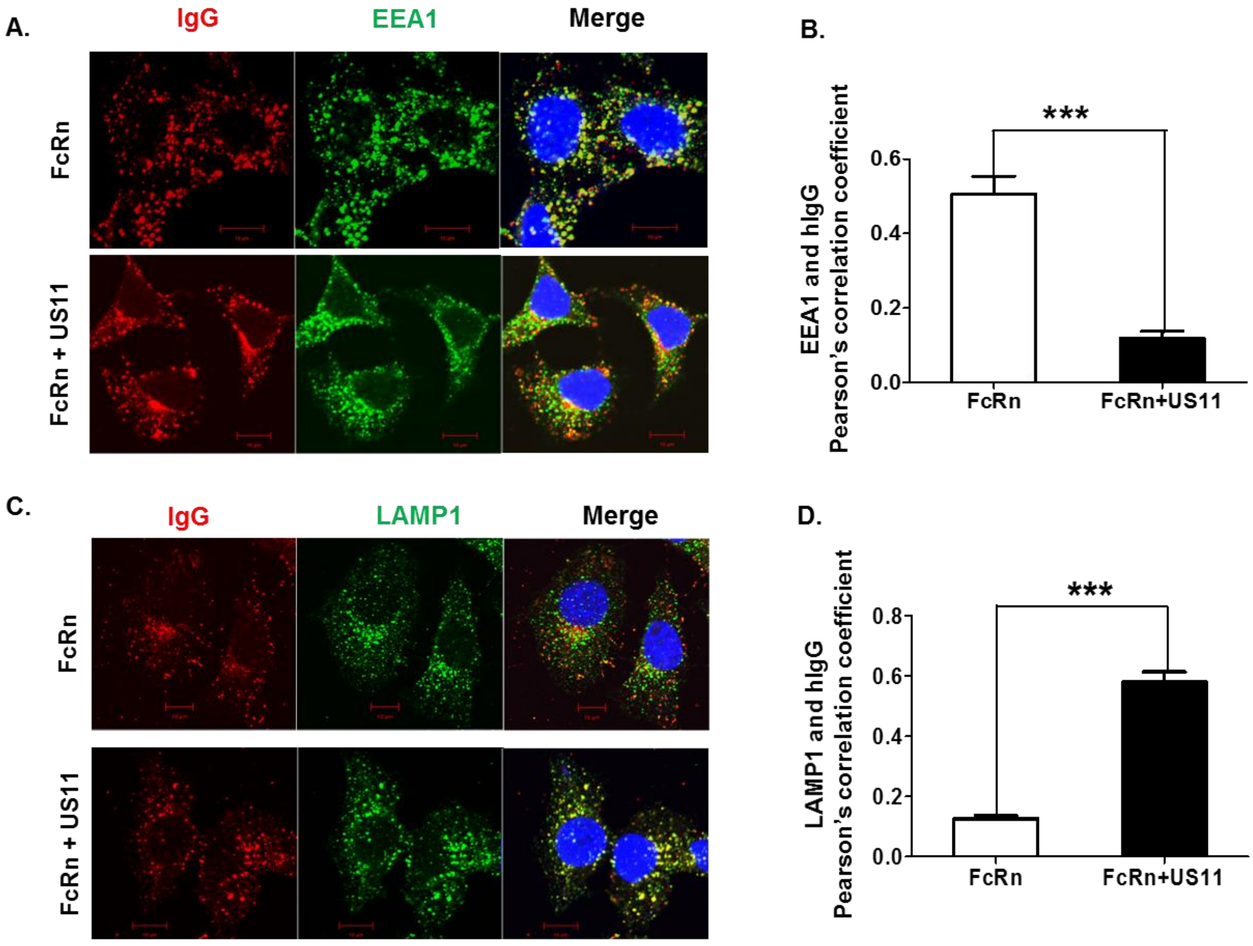

Supplementary Figure 10. Human IgG trafficking inside HeLa$^{FcRn+US11}$ and HeLa^{FcRn} cells. To visualize human IgG trafficking inside HeLa$^{FcRn+US11}$, HeLa^{FcRn} (1×10^5) cells, they were incubated with 250 µg/ml human IgG for 1 hr at 37°C. After complete washing, cells were incubated with complete medium without IgG for an additional 1 hr, then fixed and stained by immunofluorescence for the co-localization of human IgG with the early endosomal marker EEA1 (***A***) or lysosomal marker LAMP1 (***C***). Scale bar: 10 µm. For Pearson's correlation coefficiency measurement, 10 scopes, each of which contains at least 10 cells, were measured for correlation coefficiency rate (***B*** & ***D***). ***$P < 0.001$ by Student's *t*-test. Source data are provided as a Source Data file.

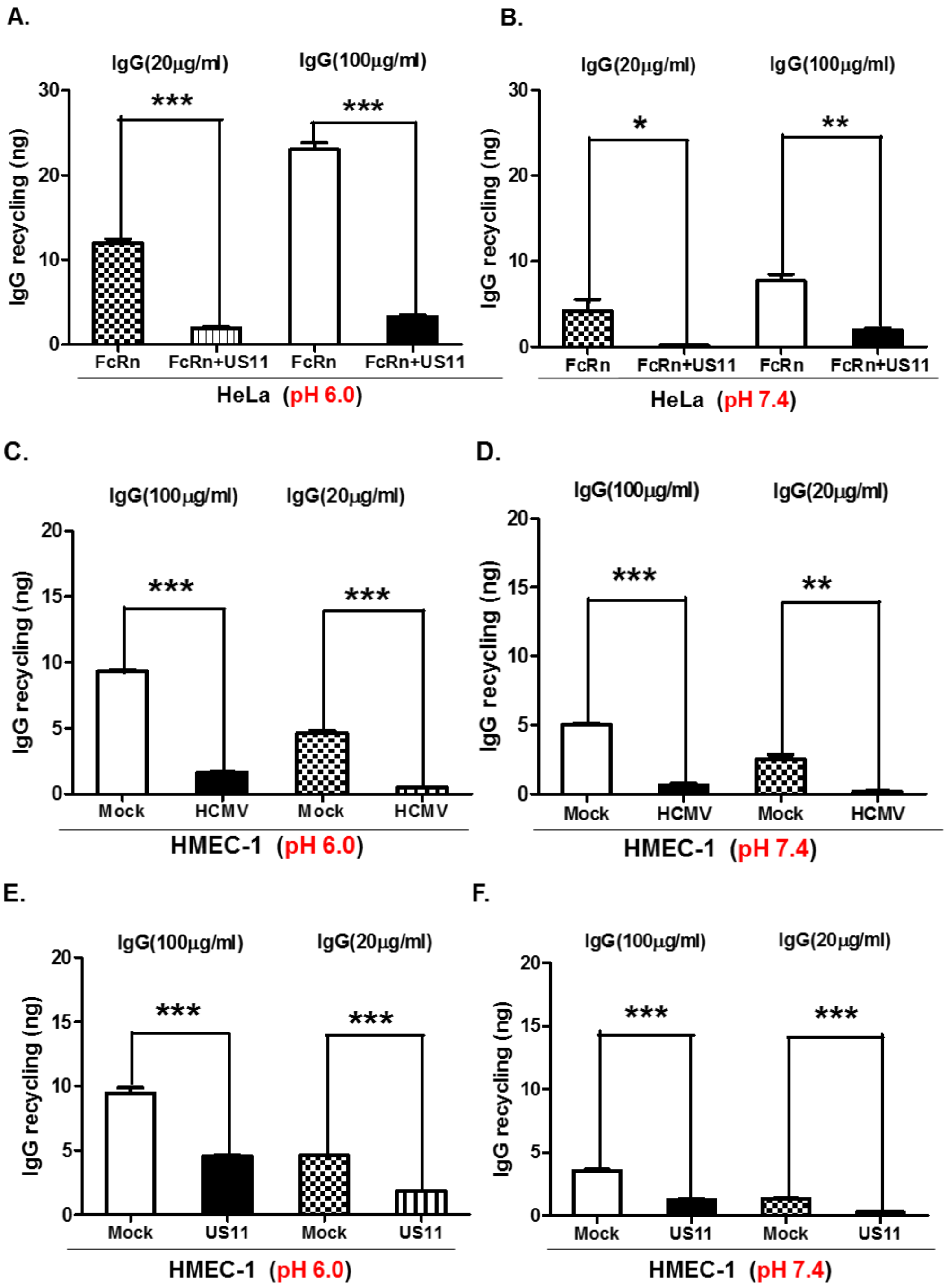

Supplementary Figure 11. Human IgG recycling is significantly reduced when cells express US11 and infected with HCMV. HeLa^{FcRn} and HeLa$^{FcRn + US11}$ cells (***A,B***), HMEC-1 cells were infected with 5 MOI of HCMV or mock-infected (***C,D***), and HMEC-1 cells were transfected with 2 μg pEF6US11 or pEF6 (mock) plasmids by Lonza Nucleofector Kit R (VCA1001) (***E,F***) and the cells were seeded in a 24 well plate (10^5 cells/well) for 48 hr. All cells were washed and starved for 1 hr in HBSS medium, and then incubated with human IgG (5 or 25 μg /250 μl) at either pH 6.0 or pH 7.4 condition for 4 hr. The cells were subsequently washed 4 times by HBSS (pH 7.4) and then incubated for additional 4 hrs at 37°C. The supernatants were sampled, and the recycled IgG was measured by ELISA. Human IgG recycling assay was performed according to a modified method (1). *P < 0.05, **P < 0.01, and ***P < 0.001 by Student's *t*-test. Source data are provided as a Source Data file.

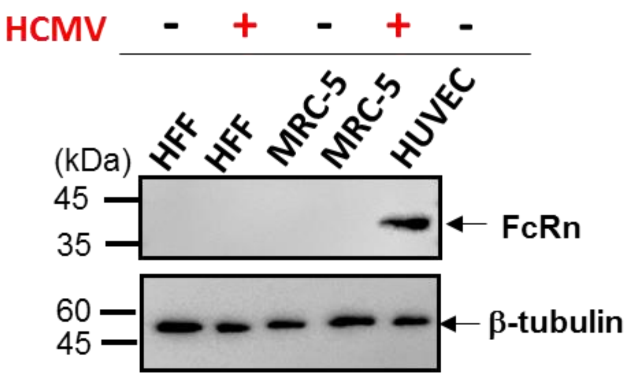

Supplementary Figure 12. Detection of FcRn expression in fibroblasts. The human foreskin fibroblasts (HFF) and fetal lung fibroblast-like MARC-5 cells were infected with HCMV at an MOI of 5. At day 2 p.i., the cell lysates (20 μg) from infected (lanes 2 & 4) or mock-infected (lanes 1 & 3) cells were subjected to Western blotting with corresponding Abs as indicated. The cell lysates (20 μg) from the HUVEC cell line (lane 5) were blotted as controls. We failed to detect FcRn protein expression in the MRC-5 and HFF cell lines by Western blot analysis; HCMV infection also did not induce FcRn expression in the MRC-5 and HFF cell lines. Both MRC-5 and HFF cell lines were originally purchased from ATCC. Source data are provided as a Source Data file.

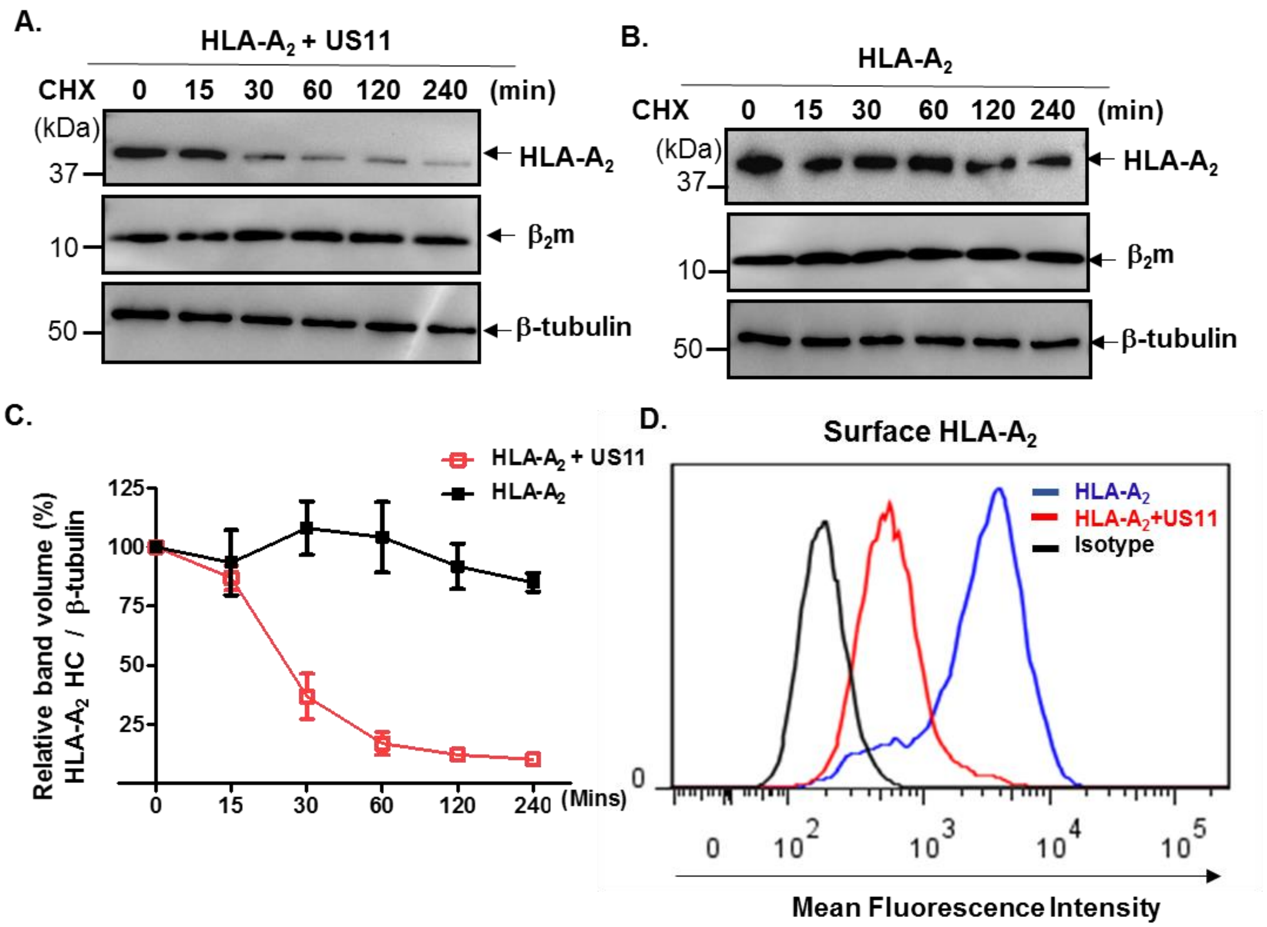

Supplementary Figure 13. US11 expression facilitates MHC class I degradation in a cycloheximide (CHX) chase assay. ***A-C.*** HeLa $^{\text{HLA-A2+US11}}$ (***A***) and HeLa $^{\text{HLA-A2}}$ (***B***) cells were treated with CHX (100 μg/ml) for the indicated time. The cells were lysed after CHX treatment and cell lysates (20 μg) were subject to Western blotting with corresponding Abs as indicated. The level of HLA-A_2 was quantified as the percentage of β-tubulin content at different time points (**C**). The percentage of time point 0 (min) is assigned a value of 100% and the values from other time points are normalized to this value. Error bars represented mean ± SEM of three independent repeats. ***D***. Cell surface expression patterns of HLA-A2 protein in the presence of US11 were measured by flow cytometry. Results are expressed as histograms of fluorescence intensity (log scale). The red or blue histograms represent staining of HeLa$^{\text{HLA-A2+US11}}$ or HeLa $^{\text{HLA-A2}}$ cells with anti-FLAG specific Ab. The black histograms represent cells stained with isotype-matched IgG. The staining was conducted three times with similar results. The mean fluorescence intensity (MFI) is shown on the x-axis, and the relative cell number on the y-axis. Source data are provided as a Source Data file.

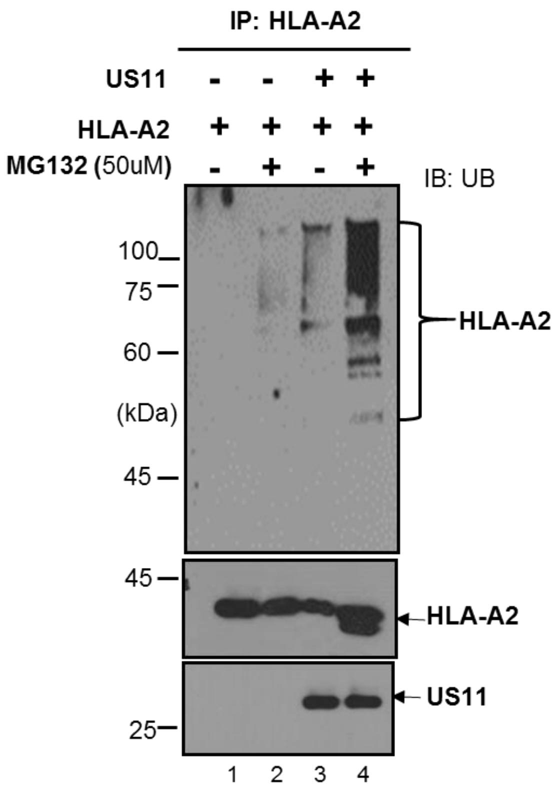

Supplementary Figure 14. MHC class I is ubiquitinated in the presence of US11 expression and MG132 treatment**.** The HeLa $^{HLA\text{-}A2+US11}$and HeLa $^{HLA\text{-}A2}$ cells were treated with proteasome inhibitor MG132 (50 μM) for 2 hr, as indicated. The HeLa $^{HLA\text{-}A2+US11}$and HeLa $^{HLA\text{-}A2}$ cells were lysed. The cell lysates (0.5 mg) were immunoprecipitated with mAb anti-FLAG for HLA-A2. The immunoprecipitates were subjected to the electrophoresis and immunoblotting analysis to detect ubiquitin and the target proteins HLA-A2 or US11, as indicated. Source data are provided as a Source Data file.

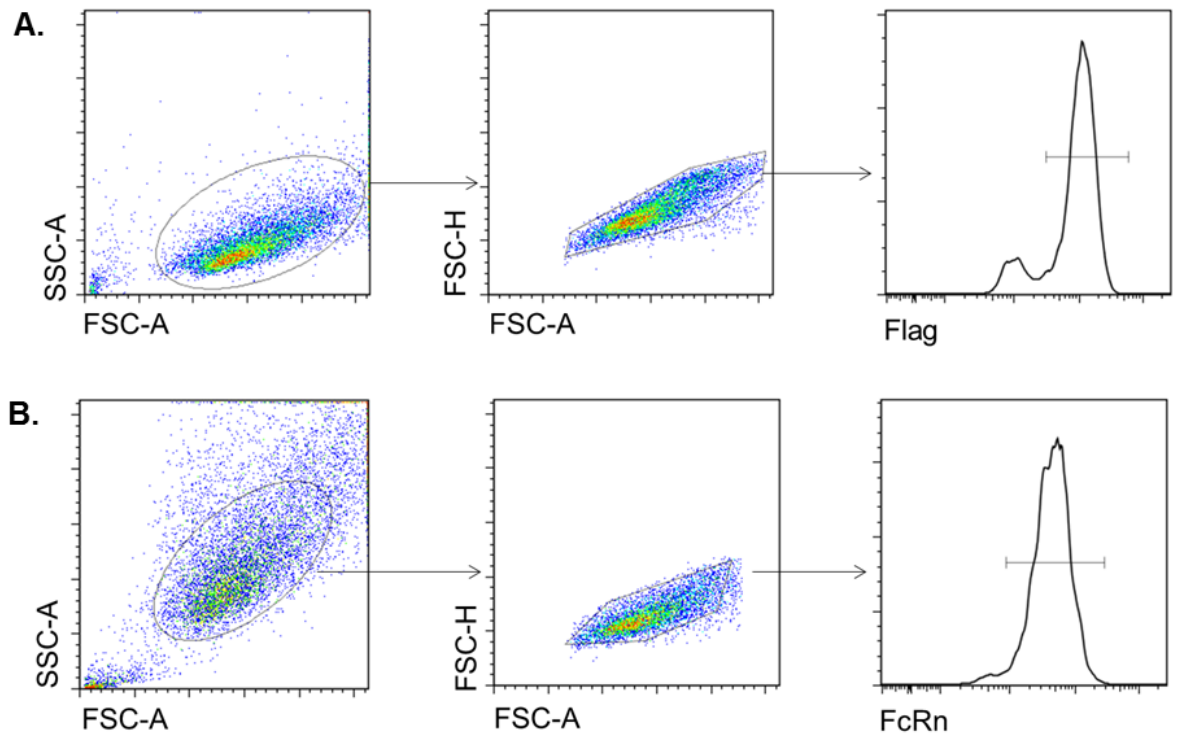

Supplementary Figure 15. Gating strategies used for flow cytometry. ***A.*** Gating strategy for detection of FLAG-FcRn (**Fig. 3*A***) , FLAG-HFE (**Fig. 3*B***) or FLAG-HLA-A_2 (**Supplementary Fig. 13*D***) expressed HeLa cells. ***B.*** Gating strategy for detection of native FcRn in HCMV- or Mock- infected THP-1 cells (**Supplementary Fig. 6*A***) and HMEC-1 cells (**Supplementary Fig. 6*B***).

Supplementary Table 1. PCR primers used in this study

Genes	Forward Primer	Reverse Primer
US11 RT (1-675)	5′-GCTCGGATCCGCCACCATGAACCTTGTAATGCTTATTC-3′	5′-GCCCTCTAGACTACCACTGGTCCGAAAACATCCAGG-3′
US11-HA	5′-GTAGTCTGGCACATCATATGGGTATAATTCAGGCATACTACCCGCGAC-3′	5′-CCATATGATGTGCCAGACTACGCATCCTTGACTCTTTTCGATGAAC-3′
US11 Q192L	5′-CAAAACACTAGAATCACTGCCACCATCATCAGCGTATACTGCGCCGAC-3′	5′-CAGTGATTCTAGTGTTTTGGGGGCTGTATGTGAAAGGTTGGCTG-3′
GST-US11(93-531)	5′-GCGTGGATCCTTGGTGGAGACGGAGCCGTTACCGCCTC	5′-TTATGCGGCCGCCGTGAGCGCGTAGTACGCCATTAGAC-3′
US2 RT (1-600)	5′-ATGAACAATCTCTGGAAAGCCTGG	5′-TCAGCACACGAAAAACCGCATCCAC-3′
US2-HA	5′-ACGCGGATCCGCCACCATGAACAATCTCTGGAAAGC-3′	5′-GCGTCTAGACTATGCGTAGTCTGGCACATCATATGGGTAGCACACGAAAAACCGCATCCACATC-3′
HFE (67-1035)	5′-GTCGAAGCTTCGCTTGCTGCGTTCACACTCTCTG-3′	5′-ACCGTCTAGACTACTCACGTTCAGCTAAGACGTAGTG-3′
HLA-A2 (1-1098)	5′-GTCGAAGCTTGGCTCTCACTCCATGAGGTATTTCTTCAC-3′	5′-ACCGTCTAGACTACACTTTACAAGCTGTGAGAGACAC -3′
FcRn CT-/-(24-323)	5- CTTGCGGCCGCGGCAGAAAGCCACCTCTCCCTCCTG-3′	5′-ATCCTCTAGATTACCTTCTCCACAACAGAGCTCCTCC-3′
FcRn-365A-/-(24-364)	5′-CTTGCGGCCGCGGCAGAAAGCCACCTCTCCCTCCTG-3′	5′-ATCCTCTAGATTAGGTGGCTGGAATCACATTTACATC-3′
Derlin-1 RT (1-756)	5′-ATGTCGGACATCGGAGACTGGTTC-3′	5′-TCACTGGTCTCCAAGTCGAAAGCC-3′
Derlin-1 (1-756)	5′-GACGTCTAGAGCCACCATGTCGGACATCGGAGACTGGTTC-3′	5′-GAATCTCGAGTCACAGATCCTCTTCTGAGATGAGTTTTTGTTCCTGGTCTCCAAGTCGAAAGCC-3′
Derlin-1 CT-/-(1-525)	5′-CAGGAACAGAAGCTCATCTCAGAAGAGGATCTGAATAG-3′	5′-AGCTTCTGTTCCTGGTCTCCCAGATGTCCAACCAGATTTCCAATAAG-3′
Derlin-1 NT-/-(66-756)	5′-AGCCACCATGGTCGCCGTGCCCTTGGTCGGCAAACTC-3′	5′-GCACGGCGACCATGGTGGCTCTAGCCAGCTTGGGT-3′

Supplementary Table 2. List of cell lines used in this study

Cell line	Proteins expressed	Expression vector	Selection
HeLa ^{FcRn}	Flag-tagged FcRn	pCDNA-Flag	G418
HeLa $^{FcRn\ CT-/-}$	Flag-tagged FcRn cytoplasmic tailless mutant	pCDNA-Flag	G418
HeLa $^{FcRn\ 365A-/-}$	Flag-tagged FcRn C-terminal alanine deletion mutant	pCDNA-Flag	G418
HeLa^{US11}	HA-tagged US11	pEF6	Blasticidin
HeLa$^{FcRn+US11}$	Flag-tagged FcRn + HA-tagged US11	pCDNA-Flag + pEF6	G418 + Blasticidin
HeLa^{US11*}	HA-tagged US11 Q192L mutant	pEF6	Blasticidin
HeLa$^{FcRn+US11*}$	Flag-tagged FcRn + HA-tagged US11Q192L mutant	pCDNA-Flag + pEF6	G418 + Blasticidin
HeLa^{US2}	HA-tagged US2	pEF6	Blasticidin
HeLa$^{FcRn+US2}$	Flag-tagged FcRn + HA-tagged US2	pCDNA-Flag + pEF6	G418 + Blasticidin
HeLa^{HFE}	Flag-tagged HFE	pCDNA-Flag	G418
HeLa$^{HFE+US11}$	Flag-tagged HFE + HA-tagged US11	pCDNA-Flag + pEF6	G418 + Blasticidin
HeLa^{HLA-A2}	Flag-tagged HLA-A2	pCDNA-Flag	G418
HeLa$^{HLA-A2+US11}$	Flag-tagged HLA-A_2 + HA-tagged US11	pCDNA-Flag + pEF6	G418 + Blasticidin
HeLa$^{Derlin-1}$	Myc-tagged Derlin-1	pSecTag2/Hygro A	Hygromycin
HeLa$^{FcRn+Derlin-1}$	Flag-tagged FcRn + Myc-tagged Derlin-1	pCDNA-Flag + pSecTag2/Hygro A	G418 + Hygromycin
Caco-2^{US11}	HA-tagged US11	pEF6	Blasticidin
Caco-2^{Vector}		pEF6	Blasticidin
HFF ^{FcRn}	Flag-tagged FcRn	pCDNA-Flag	G418

Supplementary Table 3. siRNA primers used in this study

Genes		SiRNAs
TMEM129	#1	5′-AUAGGCGAGAGUGAAGGUC-3′
TMEM129	#2	5′-ACCGGUGGCAAACUUGUCA-3′
US11	#1	5′-AGUCCCGGAGCCAFUAGCGUU-3′
US11	#2	5′-UCGCACUCUACAUAAUAAGUU-3′
Ube2j1		5′-UUAUUUGCCUAGCCAGUUC-3′
Ube2j2		5′-UUGAACCACUCGAGAAUAU-3′

Supplementary Reference

1. SGrevys A, et al. A human endothelial cell-based recycling assay for screening of FcRn targeted molecules. Nat Commun. 2018; 9:621. doi: 10.1038/s41467-018-03061-x.
