# Supplementary material for: Human cytomegalovirus evades antibody-mediated immunity through endoplasmic reticulum-associated degradation of the FcRn receptor
Source: Nat Commun. 2019 Jul 9;10:3020. doi: 10.1038/s41467-019-10865-y (PMC6617459; doi:10.1038/s41467-019-10865-y)
Supplement: Supplementary file 3 — Reporting Summary [file 41467_2019_10865_MOESM3_ESM.pdf]

nature research

Corresponding author(s): Xiaoping Zhu
Last updated by author(s): Apr 15, 2019

Reporting Summary

Nature Research wishes to improve the reproducibility of the work that we publish. This form provides structure for consistency and transparency in reporting. For further information on Nature Research policies, see Authors & Referees and the Editorial Policy Checklist.

Statistics

For all statistical analyses, confirm that the following items are present in the figure legend, table legend, main text, or Methods section.

n/a	Confirmed	
☐	☒	The exact sample size (*n*) for each experimental group/condition, given as a discrete number and unit of measurement
☐	☒	A statement on whether measurements were taken from distinct samples or whether the same sample was measured repeatedly
☐	☒	The statistical test(s) used AND whether they are one- or two-sided<br>*Only common tests should be described solely by name; describe more complex techniques in the Methods section.*
☐	☒	A description of all covariates tested
☒	☐	A description of any assumptions or corrections, such as tests of normality and adjustment for multiple comparisons
☐	☒	A full description of the statistical parameters including central tendency (e.g. means) or other basic estimates (e.g. regression coefficient) AND variation (e.g. standard deviation) or associated estimates of uncertainty (e.g. confidence intervals)
☐	☒	For null hypothesis testing, the test statistic (e.g. *F*, *t*, *r*) with confidence intervals, effect sizes, degrees of freedom and *P* value noted<br>*Give P values as exact values whenever suitable.*
☒	☐	For Bayesian analysis, information on the choice of priors and Markov chain Monte Carlo settings
☒	☐	For hierarchical and complex designs, identification of the appropriate level for tests and full reporting of outcomes
☐	☒	Estimates of effect sizes (e.g. Cohen's *d*, Pearson's *r*), indicating how they were calculated

Our web collection on statistics for biologists contains articles on many of the points above.

Software and code

Policy information about availability of computer code

Data collection	Image Lab 5.2, Wallac 1420 Manager, Zeiss LSM 510 ZEN 2009, BD FACSDiva
Data analysis	GraphPad Prism 5, Microsoft Excel, Zeiss LSM Image Browser, Microsoft Powerpoint, Flowjo

For manuscripts utilizing custom algorithms or software that are central to the research but not yet described in published literature, software must be made available to editors/reviewers. We strongly encourage code deposition in a community repository (e.g. GitHub). See the Nature Research guidelines for submitting code & software for further information.

Data

Policy information about availability of data

All manuscripts must include a data availability statement. This statement should provide the following information, where applicable:

- Accession codes, unique identifiers, or web links for publicly available datasets
- A list of figures that have associated raw data
- A description of any restrictions on data availability

Yes, We have all data available along with raw data.

Field-specific reporting

Please select the one below that is the best fit for your research. If you are not sure, read the appropriate sections before making your selection.

☒ Life sciences ☐ Behavioural & social sciences ☐ Ecological, evolutionary & environmental sciences

For a reference copy of the document with all sections, see nature.com/documents/nr-reporting-summary-flat.pdf

Life sciences study design

All studies must disclose on these points even when the disclosure is negative.

Sample size	All experiments and analysis were performed for at least three times. All necessary required controls were included in our experiments. Time course experiments were also performed to verify our hypothesis.
Data exclusions	Rationale for data exclusion is the data which cannot be reproduced because of technical and reagent failures.
Replication	All key experiments and analysis were performed for at least three times. All necessary required controls were included in our experiments.
Randomization	All samples allotted for experiments were treated randomly without any preference.
Blinding	All key experiments were performed blindly by other investigators.

Reporting for specific materials, systems and methods

We require information from authors about some types of materials, experimental systems and methods used in many studies. Here, indicate whether each material, system or method listed is relevant to your study. If you are not sure if a list item applies to your research, read the appropriate section before selecting a response.

Materials & experimental systems

n/a	Involved in the study	
☐	☒	Antibodies
☐	☒	Eukaryotic cell lines
☒	☐	Palaeontology
☒	☐	Animals and other organisms
☒	☐	Human research participants
☒	☐	Clinical data

Methods

n/a	Involved in the study	
☒	☐	ChIP-seq
☐	☒	Flow cytometry
☒	☐	MRI-based neuroimaging

Antibodies

Antibodies used

Mouse anti- Flag antibody (clone M2), Sigma-Aldrich, Cat.F1804-50UG. Lot#SLBW5142
Rat Anti-Flag antibody (clone L5), Biolegend, Cat.637301. Lot#B227795
Rabbit anti-Flag antibody, Sigma-Aldrich, cat. F7425. Lot# 122M4795
Rat Anti-HA antibody (clone 3F10), Roche, cat.11867423001. Lot#11608200
Mouse anti-Myc antibody (clone 9B11), Cell Signaling, Cat. 2276S Lot# 24
Rabbit anti-Myc antibody (clone 71D10), Cell Signaling, Cat. 2278T
Mouse Anti-FcRn antibody (clone B-8), Santa Cruz Biotechnology, cat. Sc-271745. Lot# F1516
Rabbit anti-beta 2 microglobulin antibody (EP2978Y), Abcam, Cat. ab75853
Rabbit anti-beta tubulin antibody, Sigma-Aldrich Cat.T2200-200UL. Lot#127K4815
Mouse anti-PP65 (clone 3A12), Abcam, Cat. ab6503, Lot# GR185391-5
Rabbit anti- PP65 rbiorbyt, Cat. orb10511.
Mouse Anti-Ubiquitin antibody (clone P4D1), Santa Cruz Biotechnology, cat. Sc-8017.
Rabbit anti-TMEM129, Sigma-Aldrich, cat. SAB1302253.
Mouse anti-MHC class I (clone W6/32), Enzo Life Sciences, cat. ALX-805-711-C100
Rabbit anti-TfR1 (CD71), Santacruz Biotechnology, Cat. sc-9099. Lot # E1313.
Mouse anti-Ube2j1 (clone B-6), Santa Cruz Biotechnology, Cat. sc-3777002. Lot# E3116
Rabbit anti-Ube2j2 Novus biological, Cat. NBP1-59760. Lot# QC13669
Mouse anti- EEA-1 (clone 14), BD Biosciences, Cat. 610457. Lot#4059993
Mouse anti-LAMP-1 (clone 25), BD Biosciences, Cat. 611042. Lot# 8248766
ELISA Goat anti-Human IgG Fc HRP conjugated antibody, Bethyl, Cat. A80-104P-80
ELISA Goat anti-Human IgG Fc Affinity purified antibody, Bethyl, Cat. A80-104A-8
HRP-conjugated goat anti-rat secondary antibody, Southern Biotech, Cat.3030-05. Lot#F4907-PJ20M
HRP-conjugated goat anti-rabbit secondary antibody, Southern Biotech, Cat.4030-05. Lot#g1114-TC74
HRP-conjugated goat anti-mouse secondary antibody, Southern Biotech, Cat.1010-05. Lot#C1411-N232C
HRP-conjugated Goat anti-Human IgG Fc secondary antibody, Southern Biotech, Cat.2081-05. Lot#L5311-SE25D
Alexa Fluor 555-conjugated goat anti-rabbit secondary antibody, Life Technologies, Cat. A21430. Lot#1739921.
Alexa Fluor 555-conjugated goat anti-mouse secondary antibody, Life Technologies, Cat. A21424. Lot#1802436.
Alexa Fluor 555-conjugated goat anti-human secondary antibody, Life Technologies, Cat. A21433. Lot#662495.
Alexa Fluor 488-conjugated goat anti-mouse secondary antibody, Life Technologies, Cat. A11001.
Alexa Fluor 488-conjugated goat anti-Rabbit secondary antibody, Abcam, Cat.ab150077. Lot# GR127678-1
Alexa Fluor 488-conjugated goat anti-rat secondary antibody, Life Technologies, Cat. A11006. Lot#1259366.

Validation

All antibodies purchased from commercial companies were validated by the manufacturer and we verify the quality of the antibody based on the product information provided from the company.

Antibody specific against US11 produced in our lab was verified by US11 transfected HeLa cell line and mock transfected cell line. This US11 antibody was readily available to use for others.
The mouse hybridoma 12CA5 which generates mouse anti-HA epitope and mouse hybridoma BBM1 which generates mouse anti-beta 2 microglobulin antibody were verified in our lab by HA tagged proteins.
Rabbit anttibody specific for FcRn produced in our lab was verified by previous researchers and published in journal of Immunology. (Ye, L., Liu, X., Rout, S., Li, Z., Yan, Y., Lu, L., Kamala, T., Song, W., Samal, K. S. and Zhu, X..2008.The MHC Class II-Associated Invariant Chain Interacts with the Neonatal Fcγ Receptor and Modulates Its Trafficking to Endosomal/Lysosomal Compartments. J. Immunol 181: (4) 2572-2585;)

Eukaryotic cell lines

Policy information about cell lines

Cell line source(s)	All cell lines were obtained from ATCC. HMEC-1 cell line was obtained from CDC.
Authentication	Cell lines provided by ATCC were authenticated by them. The HMEC-1 endothelial cell line was verified by endothelial cell marker by flow cytometry analysis. This HMEC-1 cell line was verified by other investigators.
Mycoplasma contamination	Mycoplasma detection is performed by commercially available mycoplasma detection kit.
Commonly misidentified lines (See ICLAC register)	We do not use any commonly misidentified cell lines registered with ICLAC.

Flow Cytometry

Plots

Confirm that:

☒ The axis labels state the marker and fluorochrome used (e.g. CD4-FITC).

☒ The axis scales are clearly visible. Include numbers along axes only for bottom left plot of group (a 'group' is an analysis of identical markers).

☐ All plots are contour plots with outliers or pseudocolor plots.

☒ A numerical value for number of cells or percentage (with statistics) is provided.

Methodology

Sample preparation	HeLa, THP-1, HMEC-1 cell lines were used for flow cytometry
Instrument	BD-FACS Aria II cell sorter 643178
Software	BD FACSDiva software was used for data collection. Flowjo software was used for data analysis.
Cell population abundance	Cell sorting was not used in our studies.
Gating strategy	We performed the initial gating strategy with unstained, isotype control, and specific antibody treated samples along with positive and negative controls. We have provided a supplementary figure exemplifying the gating strategy.

☒ Tick this box to confirm that a figure exemplifying the gating strategy is provided in the Supplementary Information.
